# Optimization frameworks for bespoke sensory encoding in neuroprosthetics

**DOI:** 10.1063/5.0249434

**Published:** 2025-05-20

**Authors:** Franklin Leong, Silvestro Micera, Solaiman Shokur

**Affiliations:** 1 Translational Neural Engineering Laboratory (TNE Lab), Neuro-X Institute, EPFL, Geneva, Switzerland; 2 Modular Implantable Neurotechnologies (MINE) Laboratory, Università Vita-Salute San Raffaele and Scuola Superiore Sant'Anna, Milan, Italy; 3 Bioelectronics and Bioengineering Area, The BioRobotics Institute and Department of Excellence in Robotics and AI, Scuola Superiore Sant'Anna, Pisa, Italy; 4 Department of Clinical Neurosciences, University Hospital of Lausanne and University of Lausanne, Lausanne, Switzerland

## Abstract

Restoring natural sensation via neuroprosthetics relies on the possibility of encoding complex and nuanced information. For example, an ideal brain–machine interface with sensory feedback would provide the user with sensation about movement, pressure, curvature, texture, etc. Despite advances in neural interfaces that allow for complex stimulation patterns (e.g., multisite stimulation or the possibility of targeting a precise neural ensemble), a key question remains: How can we best exploit the potential of these technologies? The increasing number of electrodes coupled with more parameters being explored leads to an exponential increase in the number of possible combinations, making a brute-force approach, such as systematic search, impractical. This Perspective outlines three different optimization frameworks—namely, the explicit, physiological, and self-optimized methods—allowing one to potentially converge faster toward effective parameters. Although our focus will be on the somatosensory system, these frameworks are flexible and applicable to various sensory systems (e.g., vision) and stimulator types.

## I. INTRODUCTION

In recent years, many noninvasive and implantable strategies have been proposed to restore a variety of sensations (e.g., vision,^1^ tactile,^2^ auditory^3^). For example, somatosensory neuroprosthetics have been developed for upper limb,^4–6^ lower limb,^7,8^ stroke,^9^ spinal cord injury^10^ with promising outcomes. Raspopovic *et al.* restored the sensation of force, stiffness, and shape in arm amputees using transversal intrafascicular multichannel electrodes (TIME),^4^ while Oddo *et al.* demonstrated the discrimination of different textures using microneurography and TIME.^11^ For noninvasive options, D'Anna *et al.* managed to utilized transcutaneous electrical nerve stimulation (TENS) to restore force feedback.^12^ Several studies with brain implants showed somatosensory restoration via intracortical microstimulation (ICMS) in both non-human primate^13,14^ and humans.^8^ Recently, the field has also begun to gravitate toward restoration of thermal sensation.^15–18^

Sensory encoding refers to the process in which information from the environment is captured and converted into neuronal signals that are interpreted by the brain. The loss of sensory organs disrupts the biological sensory encoding pathways. However, an artificial sensor could replace the sensory organs.^15,19^ Coupled with neural interfaces that map the input from the sensor to stimulation (e.g., electrical, mechanical, thermal) of the nervous system, this allows for artificial restoration of the sensory encoding process.^4,5,15,16,20,21^

Although lost sensations can be partially restored through sensory encoding, it poses a fundamental challenge due to the subjective nature of personal perceptions. This is in contrast to the relatively more objective task of reading and decoding neural signals for motor neural prosthesis.^22,23^ This concept of the subjective nature of individual perceptions is also known as “qualia”—exemplified by the idea that the personal experience of “what is red?” or “feeling pain” is hard to communicate and unique. The challenge in sensory restoration arises from the variability of the qualia, influenced by factors such as prior sensory experiences and neural plasticity. In addition, the placement of the neural interface inevitably varies between users, contributing to the elicitation of different perceptions.

The inherent subjectivity of sensory perception complicates the evaluation of stimulation strategies, making it challenging to determine their effectiveness. To mitigate this, researchers have developed objective assessment methods that minimize bias. For instance, Iberite and colleagues employed a matching task where artificial sensations from prosthetics were presented alongside reference natural sensations, allowing users to indicate whether they matched^15^—this is less susceptible to bias compared to asking participants to describe how the artificial sensation felt. Another strategy involves assessing the impact of sensory feedback on motor performance, such as moving blocks with varying thermal^18^ or fragility levels,^5^ as well as measuring the time taken to reach for an object.^24^ Finally, embodiment assessments serve as proxies for evaluating the efficacy of sensory feedback. Methods such as the cross-congruency paradigm^25^ and peripersonal space tests^26^ help determine the extent to which a prosthetic limb is integrated into the body schema. Together, these objective approaches offer a more comprehensive and reliable evaluation of sensory feedback in neuroprosthetics.

**FIG. 1.**
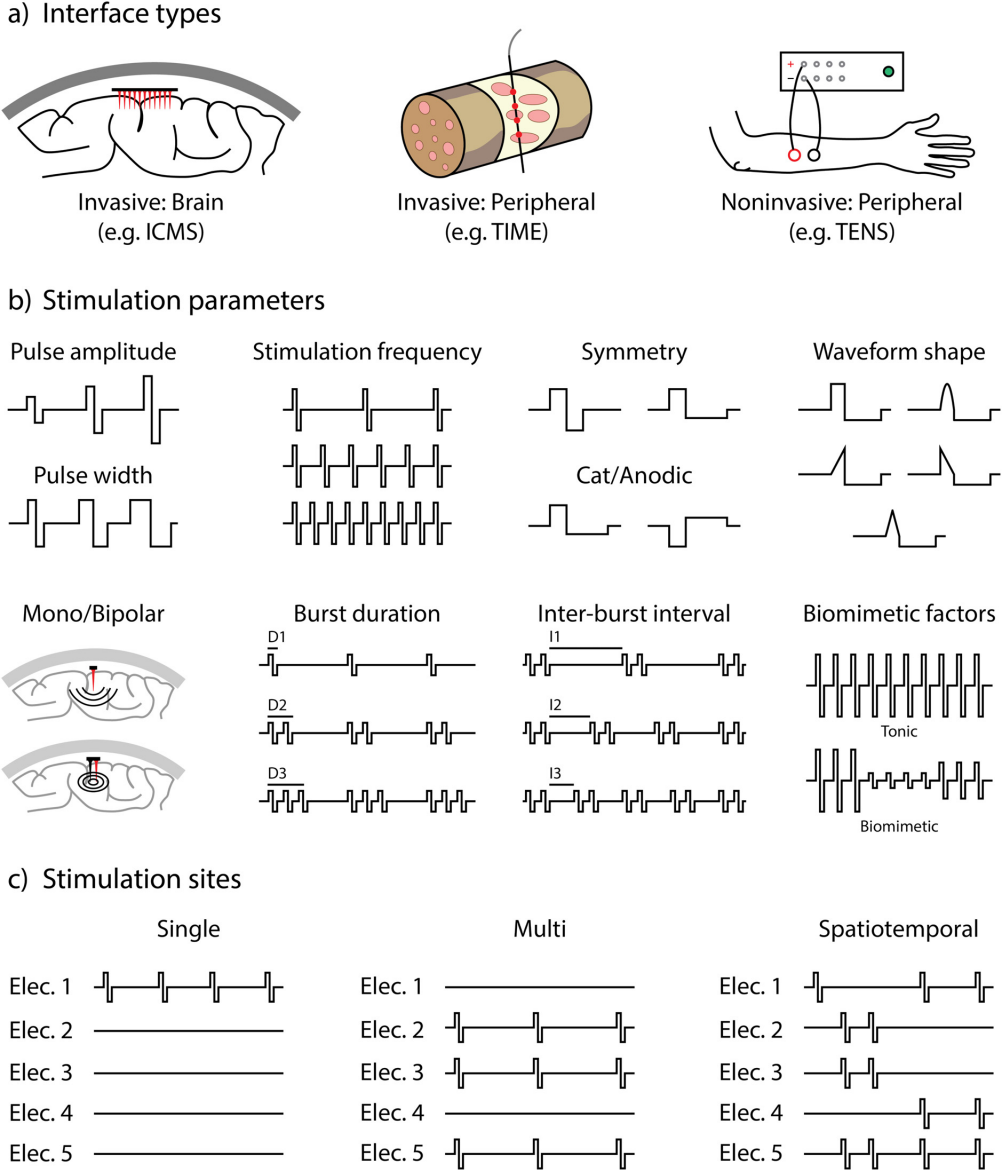
Sensory encoding of neural interface. (a) Examples of different types of neural interfaces. (b) Possible stimulation parameters that can be optimized. (c) Possible ways of selecting the stimulation sites.

Beyond the evaluation of stimulation strategies, subjectivity also poses a fundamental challenge in optimizing stimulation parameters. A key question in sensory feedback is how to best encode sensory information through artificial stimulation to produce meaningful and naturalistic perceptions. Finding an effective set of parameters involves navigation in a very large search space. For example, considering ICMS of the somatosensory cortex [Fig. 1(a)], two categories of stimulation parameters could be explored: the stimulation parameters [Fig. 1(b)] and the stimulation sites [Fig. 1(c)]. Typically, stimulation parameters such as pulse amplitude, pulse width, and stimulation frequency were modulated. Waveform shape, symmetry, cathodic or anodic first, and the use of mono/bipolar stimulation have also been investigated. Furthermore, in recent years, biomimetic patterns have been introduced.^27,28^ Biomimetic stimulation is a complex form of stimulation that mirrors the encoding methods utilized by the nervous system in nature. In addition to biomimicry, stimulation bursts have also been used and can be defined by parameters such as burst duration and interburst interval.^29^

When considering the stimulation sites, we can generally select a single electrode or multiple electrodes [Fig. 1(c)]. The stimulation parameters are usually similar across the electrodes. Nevertheless, the discipline is increasingly focusing on investigating the spatiotemporal characteristics of neurons during stimulation.^30^ Initially, neuronal activity is captured through implants as specific stimuli are administered. Subsequently, they stimulate the implanted region guided by the recorded neuronal activity. For instance, one group of neurons may become active in the initial phase, whereas a different group might activate subsequently.

Most, if not all, of the parameters discussed are mutually exclusive; as such, this leads to an exponential increase in the number of possible combinations across the different parameters. Studies of computational models^8^ and animal models^31,32^ have helped narrow down this huge multidimensional search space. Nevertheless, to unlock the next tier of bespoke sensory encoding—encompassing more natural sensation, precise, and stable somatotopy—we need faster or automated methods to optimize the parameters, exploiting the extensive possibilities offered by the multiparameter and multisite stimulation.

To this end, in this Perspective, we first discuss the conventional approach of developing sensory encoding strategies, which involves devising encoding method with novel strategy or modality. Then, we elaborate on the three different optimization frameworks—explicit, physiological, self-optimized—for efficient optimization of stimulation parameters for the different sensory encoding strategies already explored through the conventional approach. Although we focused on the somatosensory system, these frameworks are designed to be adaptable across different sensory systems and types of stimulators. Finally, we explore how the different optimization frameworks could work in tandem.

## II. CONVENTIONAL APPROACH

Traditionally, the development of sensory encoding strategies has been guided by theoretical models.^33–39^ More recently, there has also been an increase in developmental efforts inspired by biological mechanisms observed in natural sensory processing.^8,40–43^ Researchers identify potential stimulation parameters, such as those illustrated in Fig. 1(b), to encode sensory information. Then, these parameters are validated on human subjects through experimental trials.^11,12,29,44^ The effectiveness of these parameters is often assessed through post-stimulation psychophysical tests and post-experiment questionnaires, where subjects describe their sensory experiences, including aspects such as intensity, quality, and spatial localization.

Sensory encoding strategies can vary in complexity. For example, in tactile sensory feedback, early efforts focused on a simple mapping of force sensor values to the intensity of stimulation parameters.^45^ Then, apparent moving sensation could be elicited through sequential stimulation of multiple locations.^46^ More recently, complex stimulation patterns inspired by biomimicry have shown a marked improvement in the naturalness of the elicited tactile sensation. Saal and Bensmaia proposed a biomimetic approach to stimulate the peripheral nerve in 2015,^27^ where they subsequently released a biophysical model.^28^ Valle *et al.* validated the approach in multiple studies in which they based the stimulation parameters on the output of the biophysical model. This elicited a sensation in amputees that felt more natural compared to that elicited by a more naive method.^5,8^

Beyond increasing the complexity of stimulation, sensory encoding can be expanded by incorporating additional modalities. For example, rather than solely providing pressure or vibration feedback, researchers have explored thermal feedback to enhance the range of sensory information conveyed to users.^15,16,18^ Introducing new modalities can also unlock previously unattainable possibilities, such as encoding wetness sensations through thermal feedback.^17^

## III. OPTIMIZATION FRAMEWORKS

**FIG. 2.**
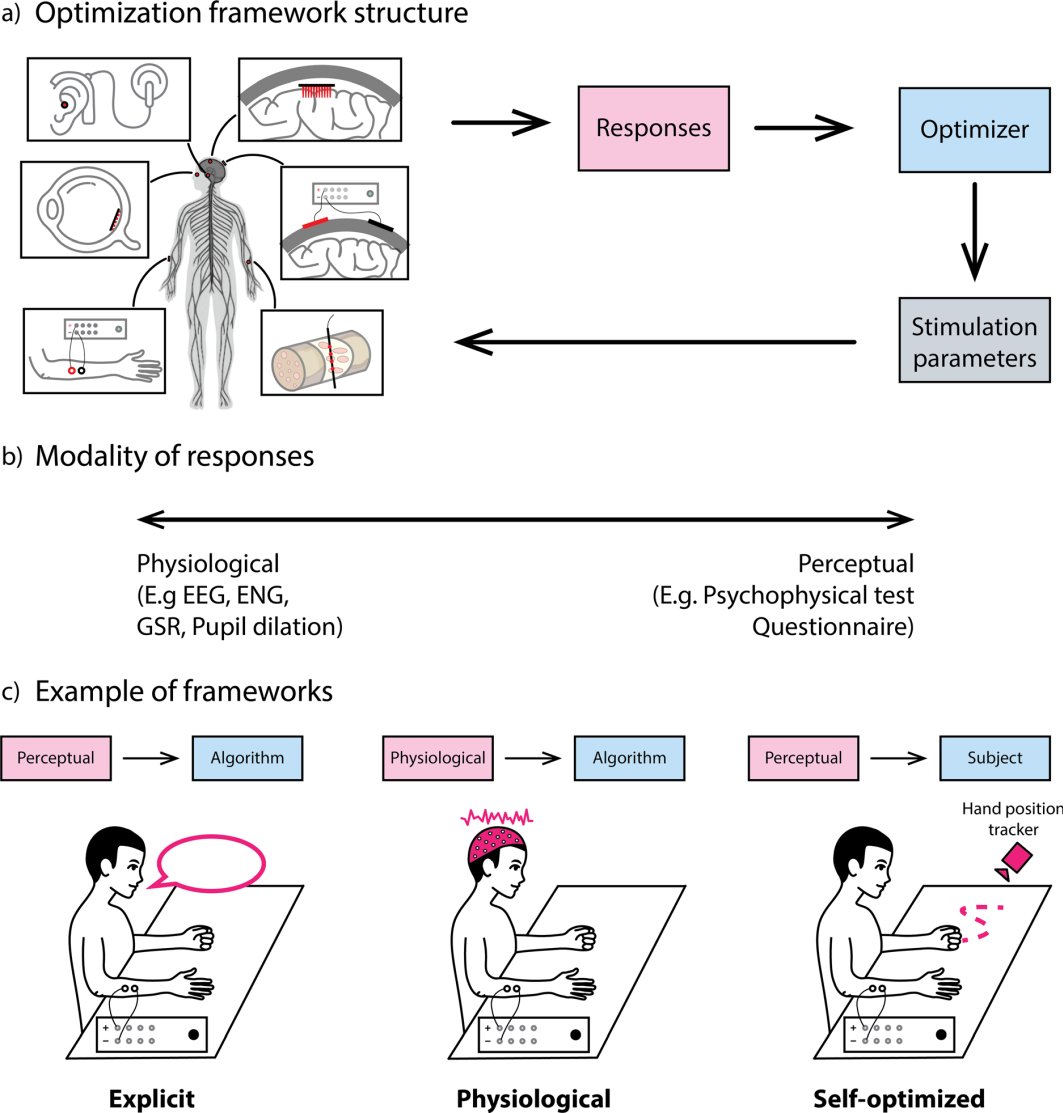
Frameworks for sensory encoding optimization. (a) Skeletal structure of a generic optimization framework. (b) Possible modality of responses that can be recorded. (c) Example of different frameworks depending on the combination of types of response and optimizer.

Although the conventional approach is effective for identifying potential sensory encoding strategies, eliciting a bespoke sensation requires optimization. For instance, one might propose that a burst-stimulation approach is suitable for sensory encoding, but to maximize its potential, various parameters should be tuned. Optimization frameworks can be employed to fine-tune these parameters to elicit the desired sensation. Figure 2 highlights the different types of optimization framework. In general, the main components of an optimization framework consist of responses, optimizer, stimulator, and its stimulation parameters [Fig. 2(a)]. This skeletal framework is applicable to different types of stimulation, ranging from invasive/noninvasive, brain/peripheral nerve stimulation, or even retinal and auditory stimulation. Upon stimulation, responses could be acquired. Such responses can be categorized into physiological or perceptual [Fig. 2(b)]. Examples of physiological signals could be electroencephalogram (EEG) or electroneurography (ENG), while the most common measure of perceptual information is through psychophysical tests. The output of the responses is then relayed to an optimizer that decides on the stimulation parameters of the next iteration, closing the optimization loop. The end point of the optimization process is contingent on factors such as the maximum number of iterations or a specified time constraint.

The crux of an optimization framework lies in the choice of responses and optimizer. Depending on the combination of responses and optimizer, we obtain three different optimization frameworks. Figure 2(c) illustrates how the different combinations generate different optimization frameworks. Briefly, when we obtain perceptual data from the subject post-stimulation and we utilize algorithms for the tuning of stimulation parameters, we have the explicit framework. Changing the responses from perceptual to physiological signals results in a physiological framework. On the other hand, if we retain perceptual data but instead change the optimizer to the subject themselves, we get the self-optimized framework. In Sec. IV–VI, we elaborate on the different responses–optimizer combinations and the pros and cons associated with each framework.

## IV. EXPLICIT

Conventionally, perceptual responses are collected through psychophysical tests or questionnaires during the experimental process to assess the effectiveness of the stimulation applied.^47–49^ This approach is logical considering that perceptual responses offer direct and valuable insights into the sensory experiences of the participants. It is therefore intuitive to utilize these perceptual responses as a means of optimization. This concept forms the basis of the explicit framework, in which perceptual responses are fed into an algorithmic optimizer as input [Fig. 2(c)].

Some example studies utilizing the explicit frameworks include Borda *et al.*, who used reinforcement learning algorithms to adjust TENS parameters for lower limb sensory feedback. Participants provided real-time feedback on the sensations experienced, allowing the system to learn and optimize stimulation settings effectively.^50^ Multiple studies also used variations of Bayesian optimizations to adapt stimulation parameters.^7,51,52^ Their method efficiently navigated the complex parameter space to identify optimal settings. In visual prosthetics, Fauvel and Chalk combined simulation with limited human feedback to optimize stimulation parameters,^53^ while Lee *et al.* presented a user preference optimization approach using RankNet for exoskeleton control, which could easily be adapted to optimize sensory feedback parameters.^54^ Another common example is cochlear implant programming. In this process, an audiologist adjusts key stimulation parameters based on user feedback. First, the audiologist determines the T-levels (Threshold levels), which represent the softest sounds the user can detect. Next, they assess the C/M-levels (Comfort or Most Comfortable levels), which define the loudest sounds that remain comfortable for the user.^55^

A key consideration in the explicit framework is the trade-off between the amount of feedback required from the subject and the number of iterations that can be performed within practical constraints. Minimizing the need for extensive subject feedback—shorter psychophysical tests—allows for more iterations and rapid data generation, which is beneficial for data-driven optimization methods such as machine learning algorithms. However, insufficient feedback could prevent the algorithm from accurately identifying the optimal stimulation parameters, as it may lack critical information about the user's subjective experience. Therefore, domain expertise remains essential in designing the optimization protocol. Experts must determine the optimal balance between the quantity and quality of information needed from the subject and the feasible number of iterations within a limited time frame, ensuring both efficiency and effectiveness in the optimization process.

The explicit framework represents a significant advancement over conventional methods by incorporating real-time human feedback into the optimization of stimulation parameters. This approach enables a more dynamic and responsive refinement process, allowing for the identification of effective parameters. However, it also introduces new challenges, particularly in the design of experiments to balance the trade-offs between the quantity of user input and the number of iterations that can be conducted.

## V. PHYSIOLOGICAL

While psychophysical tests have traditionally served as the gold standard for evaluating participants' perceptions, efforts to optimize stimulation parameters have been constrained by the speed at which individuals can complete them. In the explicit framework, the need for participants to finalize these psychophysical tests before applying the results in subsequent iterations presents a significant bottleneck in the process. In a bid to address this bottleneck, some researchers have turned to the measurement of physiological responses as proxy information for perceptions.^56–58^ The physiological framework leverages computational algorithms to dynamically adjust and refine stimulation parameters based on proxy measures that correlate with the desired sensory perception as shown in Fig. 2(c). As this method bypasses the need for human feedback, theoretically it holds the potential to allow for the maximum number of iterations for optimization.

For example, in 2022, Eldeeb and Akcakaya used EEG signals to guide the adjustment of electrical stimulation parameters to generate different texture force profiles, demonstrating the potential of EEG as a proxy for the optimization of tactile sensory feedback.^56^ Apart from EEG, Birznieks and Vickery utilized microneurography and identified the neuronal code involved in vibrotactile frequency perception. In the future, adjusting stimulation parameters based on microneurography data could further enhance frequency perception.^59,60^ Other physiological signals such as those acquired from galvanic skin response and pupil dilation can also serve as optimization targets. In visual prosthetics, surrogate models have facilitated the optimization process. Our team employed an actor–model framework to build a digital twin of the biological retina to optimize stimulation parameters for visual prosthetics, potentially enhancing the quality of perceived visual stimuli without requiring extensive subjective feedback.^57^ In deep brain stimulation for Parkinson's disease, genetic algorithms based on physiological measurements have also shown promising results.^61^ In cochlear implants, beyond determining T-levels and C/M-levels, objective measures such as Electrically Evoked Compound Action Potentials, recorded via Neural Response Telemetry, are often used to optimize stimulation parameters.^62^

The primary advantage of the physiological framework is its efficiency, enabling a highly iterative and adaptive approach that could quickly converge on optimal stimulation settings without direct human input. By utilizing proxy measurements—such as neural activity patterns, physiological responses, or behavioral indicators—the system can adjust parameters in real-time, continuously learning and improving over time. This makes the physiological framework suitable also for applications where sensory feedback needs to be fine-tuned regularly, such as in dynamic environments or with changing user conditions.

However, this framework also has limitations. A fundamental challenge is the reliance on proxy measures that may not always be perfectly correlated with subjective sensory experiences. For instance, physiological signals like EEG may provide indirect information on sensory perception but might not capture the full complexity or nuances of an individual's subjective experience. When the proxy measurement does not accurately reflect the target sensation, the optimization process potentially results in suboptimal or inconsistent sensory feedback. Therefore, the effectiveness of the closed-loop system heavily depends on the selection and accuracy of the proxy data used for optimization. Moreover, in contrast to the explicit framework where subjects need to concentrate on the perception they must report, the physiological framework lacks this requirement. Consequently, throughout the session, subjects might lose focus, which can potentially influence the physiological signal.^63^

## VI. SELF-OPTIMIZED

While the explicit framework incorporates perceptual responses as input for optimization—which could be more precise than physiological responses—it simultaneously creates a bottleneck in the iterative process. On the other hand, the physiological framework boasts the potential for rapid iterations, but the reliance on optimization based on proxy information could become problematic.

The self-optimized framework offers promise to address the key limitations of the other two frameworks by empowering the subject to control the stimulation parameters. In this approach, the subject is provided with a target perception or sensation, and they adjust the stimulation parameters themselves until the desired perception is elicited. In contrast to the explicit framework, where explicit feedback is required, the self-optimized framework does not require the participants to respond. This framework leverages the subject's intuitive understanding of their own sensory experiences. At each iteration upon stimulation, the subject perceives the sensation and determines the trajectory for the upcoming set of stimulation parameters. Throughout the process, the researcher does not receive information about the perception of the subject.

Also in contrast to the physiological framework, the self-optimized framework allows for the rapid iterations possible while still accounting for the subject's feedback, albeit in an implicit form. Shokur *et al.* demonstrate this methodology by having subjects adjust vibrator stimulation parameters to elicit sensations that matched the experience of walking on specific surfaces. Participants manipulated the parameters until the tactile feedback felt congruent with the target surface, effectively personalizing the sensory encoding.^6^ The generalizability of this approach was further tested by Verbaarschot *et al.*, who showed that allowing subjects to tune their stimulation parameters improved their ability to identify different objects during tactile exploration. By directly controlling the parameters, the subjects achieved more accurate tactile sensations, enhancing object recognition.^10^

The self-optimized framework represents an interesting approach to optimizing stimulation parameters by placing control directly in the hands of the subject. This approach bypasses the need for explicit responses, thereby maximizing the number of iterations possible within a given timeframe. By eliminating the trade-off between user input and the number of iterations, the self-optimized framework has the potential to accelerate the optimization process while ensuring that the resulting parameters closely align with the subject's sensory experience. It is worth noting that the simultaneous optimization of parameters is contingent upon the setup, and by monitoring both hands, up to six parameters (3 dimensions per hand) can be optimized. Additionally, by integrating rotations into the tracking mechanism, the number of parameters subject to optimization could be increased. However, this could add complexity, potentially diminishing the framework's effectiveness. As this framework continues to evolve, it holds enormous potential for advancing sensory encoding techniques.

## VII. COMBINATION

In Secs. IV–VI, we examined the explicit, physiological, and self-optimized frameworks independently. Each of these frameworks has their unique strengths in the optimization process but also contains limitations. Integrating these frameworks can help to mitigate the individual shortcomings, potentially leading to a more robust optimization process.

Ideally, the physiological framework holds a huge potential for optimizing stimulation parameters by allowing for rapid iterations. In practice, finding an ideal proxy measure that correlates well with the desired sensory perception is challenging; physiological signals may only partially capture the subjective experience of sensations, which can be complex and context-dependent. Integrating the physiological framework with either the explicit or self-optimized framework can help enhance these proxy measures. For instance, using EEG as a proxy, initial data could be gathered by having a group of subjects follow an explicit or self-optimized protocol, where subjects adjust parameters or provide feedback on their sensory experiences. During this process, EEG signals (or other physiological signals) can be recorded alongside the process. Once subjects perceive the desired sensation, concurrent EEG data can be analyzed to identify characteristic features that correspond well to that particular sensation. By examining EEG signals across multiple subjects, it might be possible to establish a robust map of EEG features that reliably correlate with certain sensations. These neurological markers can then be used as a proxy measure in future physiological frameworks for optimization.

Even with improved proxy measurements informed by explicit or self-optimized frameworks, it is likely that these physiological signals still may not capture all the complexities of sensory perception. To address this, a combination of frameworks can be applied sequentially to leverage the strengths of each. A practical method involves using the physiological framework for an initial broad parameter adjustment, taking advantage of its rapid and efficient nature for initial coarse tuning. This helps to narrow the parameter space. Following this initial stage, the explicit or self-optimizing framework can be utilized to refine the parameters, relying on direct user perceptual responses for greater precision. By integrating user feedback for fine-tuning, this combined approach compensates for the limitations of the physiological framework and avoids the extensive time demand of the explicit framework for preliminary tuning. In essence, this method balances the quick convergence of the physiological framework with the accuracy of the explicit framework.

The self-optimized framework, which integrates the subject's intuition and control over parameter adjustments, could similarly benefit from the combination with the explicit framework. In contrast to the other framework, the self-optimized framework requires the subject to control and determine the next stimulation parameters. However, without algorithmic guidance, users may choose parameter combinations arbitrarily or inefficiently, potentially overlooking optimal regions within the search space. To tackle this issue, the framework could periodically introduce guidance in the form of system-generated suggestions. For example, during the self-optimization process, brief psychophysical tests could periodically appear to capture the user's perceptual feedback. As more data accumulate from user responses, the system could analyze patterns to recommend parameter adjustments, directing the user's exploration toward specific areas in the parameter space where optimal results are more likely. This hybrid self-optimized approach blends subjects' intuitive adjustments with data-driven suggestions, thus ensuring a more comprehensive exploration of the parameter space while preserving users' autonomy over their sensory experience adjustments. This strategy improves both the speed and accuracy of the self-optimized framework by balancing algorithmic guidance with subject-driven exploration.

## VIII. CONCLUSION

In conclusion, this Perspective provides a comprehensive overview of various frameworks to optimize stimulation parameters in sensory encoding, a pivotal aspect of neuroprosthetics in various sensory systems, including vision, auditory, and touch. As neuroprosthetic technology continues to evolve, facilitating more intricate and refined perceptions, understanding the strengths and limitations of each framework—explicit, physiological, and self-optimized—is imperative for advancing the field. Importantly, these frameworks are not isolated approaches, but can be integrated to harness their complementary strengths. Combining them can lead to more robust and efficient sensory feedback systems, ultimately enhancing the efficacy and user experience of neuroprosthetic devices.

## Data Availability

Data sharing is not applicable to this article as no new data were created or analyzed in this study.
